# Molecular Population Genetics of Elicitor-Induced Resistance Genes in European Aspen (*Populus tremula* L., Salicaceae)

**DOI:** 10.1371/journal.pone.0024867

**Published:** 2011-09-19

**Authors:** Carolina Bernhardsson, Pär K. Ingvarsson

**Affiliations:** Umeå Plant Science Centre, Department of Ecology and Environmental Science, Umeå, Sweden; The University of Queensland, Australia

## Abstract

Owing to their long life span and ecological dominance in many communities, forest trees are subject to attack from a diverse array of herbivores throughout their range, and have therefore developed a large number of both constitutive and inducible defenses. We used molecular population genetics methods to examine the evolution of eight genes in European aspen, *Populus tremula*, that are all associated with defensive responses against pests and/or pathogens, and have earlier been shown to become strongly up-regulated in poplars as a response to wounding and insect herbivory. Our results show that the majority of these defense genes show patterns of intraspecific polymorphism and site-frequency spectra that are consistent with a neutral model of evolution. However, two of the genes, both belonging to a small gene family of polyphenol oxidases, show multiple deviations from the neutral model. The gene *PPO1* has a 600 bp region with a highly elevated K_A_/K_S_ ratio and reduced synonymous diversity. *PPO1* also shows a skew toward intermediate frequency variants in the SFS, and a pronounced fixation of non-synonymous mutations, all pointing to the fact that *PPO1* has been subjected to recurrent selective sweeps. The gene *PPO2* shows a marked excess of high frequency, derived variants and shows many of the same trends as *PPO1* does, even though the pattern is less pronounced, suggesting that *PPO2* might have been the target of a recent selective sweep. Our results supports data from both *Populus* and other species which have found that the the majority of defense-associated genes show few signs of selection but that a number of genes involved in mediating defense against herbivores show signs of adaptive evolution.

## Introduction

Plants have evolved numerous defensive mechanisms to protect themselves against biotic and abiotic stress [1], [2]. Herbivores and pathogens, in turn, respond to plant defenses by evolving counter-adaptations, that render current defenses less effective or even useless [3], [4]. This can lead to an “arms race” between plants and their enemies [3], [4]. Alternatively natural selection can favor the maintenance of multiple varieties of a defensive trait within a population, either because of negative frequency-dependent selection favoring rare alleles, or due to spatially variable selection favoring different alleles in different environments [4], [5], a model known as the “balanced polymorphism” model or the “trench-warfare model” [3].

Earlier molecular population genetic surveys of plant defense genes have shown that specialist defenses (*i.e.* defenses active against a small number of natural enemies) appear to be under stronger selection than generalist defenses (*i.e.* defenses active against a broad number of enemies) [4], [6] and that selection is more likely to maintain variation within specialist defense genes. This suggest that the type of selection on genes mediating herbivore and/or pathogen responses are experiencing, is largely dependent on whether the trait in question is effective against a broad or narrow number of natural enemies: specialist defenses are more likely to evolve in a manner consistent with the balanced-polymorphism model, while generalist defenses often show pattern of intraspecific diversity that are consistent with either a neutral model or, in rare cases, show evidence for recent positive selection, which would be consistent with an arms-race model [6].

In addition to the distinction between specialist and generalist defenses, defense responses can be classified as either constitutive or inducible, with constitutive defense being always present in a plant, whereas induced defenses are only expressed after an enemy attack has occurred [7]. Induced defenses are primed through molecular signaling pathways that allow the plants to tune their response to the type of attacker they are facing. Plant hormones such as salicylic acid (SA), jasmonic acid (JA) and ethylene (ET) are known to play important roles in the regulation of the defense response, and the effectiveness of the different pathways depends on the type of attacker the plant is facing [1]. The SA-dependent defense response is in general more active against pathogens that require a living host to complete their lifecycle (biotrophic pathogens), and SA induction is associated with the accumulation of reactive oxygen species (ROS) and diverse defense related genes, including several pathogenesis-related (PR) proteins. One of the most visible manifestation of SA is the activation of a hypersensitive response (HR) at the local site of infection that ends in programmed cell death (PCD) of surrounding cells [8]. JA is synthesized through the octadecanoid biosynthesis pathway and is only one of several bioactive compounds within the class of octadecanoids which regulates a broad spectrum of plant responses [9] and is induced by, among other things, mechanical wounding and herbivory. Induction of JA triggers a suit of defense-associated biochemical responses, such as the production of protease inhibitors and polyphenol oxidase that serve to deter the herbivore, either by slowing down its development, decreasing its reproductive success, or even repelling it from the plant [10].

Owing to their long life span and ecological dominance in many communities, forest trees are subject to the attack of a diverse array of herbivorous insects and mammals throughout their range. Forest trees have developed a large number of both constitutive and inducible defenses to deal with this suite of natural enemies [11], [12]. By virtue of the large suite of genomics tools available, members of the genus *Populus* (Salicaceae) have become the *de facto* model species for studying the effects of pathogens and herbivores, both in greenhouse and under field conditions [13]–[16]. In this paper we have used molecular population genetics methods to examine the evolution of eight wound-induced genes in European aspen, *Populus tremula*. These eight genes are all associated with defensive responses against pests and/or pathogens and have earlier been shown to be strongly up-regulated in poplars as a response to insect herbivory [17]–[20]. We analyze patterns of nucleotide diversity and divergence to detect departures from neutral expectations, with a special emphasis on evaluating the likelihood of either recurrent selective sweeps or balancing selection maintaining amino acid polymorphisms.

## Materials and Methods

### Plant material and DNA sequencing

Samples of *P. tremula* were collected from the Swedish Aspen collection (SwAsp collection), which includes 116 individuals sampled from twelve different sites throughout Sweden. The SwAsp collection has been described in detail elsewhere and for more information please refer to Luquez et al. [21]. We selected the following eight genes for study: *Allene oxide synthase (AOS5*) and *Phenylalanine-ammonia lyase (PAL1)* encode proteins that are involved in the early steps of the octadecanoid- and phenylpropanoid biosynthesis pathways, respectively [9]. *NFXL1* encodes a putative transcription factor that functions as a repressor of elicitor-induced defense responses and is involved in the crosstalk between different defensive responses associated with the SA and abscisic acid (ABA) pathways [22], [23]. Endoxylanase is a family 10 glycoside hydrolase (*GHf10*) that catalyzes the initial breakdown of xylan in cell walls and has been shown to be associated with cell wall degradation [24]. Its role in plant defense is not well understood, but other GHs, such as chitinases and glucanases, have been identified as pathogen responsive (PR) proteins [25]. C*athL* encodes a cysteine proteinase of the papain family which is induced through a hypersensitive response and takes part in the programmed cell death (PCD) of cells that surround the entry site of the pathogen infection [26]. The remaining three genes we studied are all from the polyphenol oxidase gene family (*PPO1*, *PPO2*, and *PPO3*), which codes for a group of copper-containing enzymes induced by JA that catalyses the oxidation of diphenolic compounds into quinones that cross-link or alkylate dietary proteins and thereby decrease the nutritional value for herbivorous insects or pathogens [27]–[29].

Between 11 and 27 individuals were selected to be equally distributed among the 12 sampled populations, to capture as much variation as possible present in the SwAsp collection. These individuals were sequenced for the eight genes using primers that were designed from the corresponding gene model in *P. trichocarpa* v2.0, publicly available in Phytozome (http://www.phytozome.net/poplar). The gene models used were: POPTR_0009s11280.1 (*AOS5*), POPTR_0005s18520.1 (*CathL*), POPTR_0004s17900.1 (*GHf10*), POPTR_0012s04070.1 (*NFXL1*), POPTR_0006s12870.1 (*PAL1*), POPTR_0011s10950.1 (*PPO1*), POPTR_0001s39920.1 (*PPO2*) and POPTR_0011s04710.1 (*PPO3*), respectively). Five of the genes (*AOS5*, *NFXL1*, *PPO1*, *PPO2* and *PPO3*) are intronless, whereas *PAL1* has two exons and C*athL* and GHf10 have four exons each. An effort was made to sequence the entire coding region of all genes, but this was not achieved for *GHf10*, *PPO2*, and *PPO3*. For *GHf10* this was due to a region of 500 bp of missing data in the middle of the gene, which was amplified in two, non-overlapping pieces. For *PPO2* and *PPO3*, low quality sequence data at the 3′ and 5′ ends prevented us from obtaining the entire coding region of these genes. Sequencing of *AOS5*, C*athL*, *NFXL1*, *PAL1* and *PPO1* yielded the entire coding region and short sequences of the 3′ and 5′ UTRs. The length of the sequenced regions ranged from 1035 bp for GHf10 to 3454 bp for *NFXL1* (Table 1) and the length of the coding regions ranged from 594 for GHf10 to 3336 bp for *NFXL1* (Table 1).

**Table 1:** Summary statistics of eight inducible defense genes and a genome-wide average calculated over 76 reference genes.

Locus	N	Sites	Coding	S	π_tot_	π_syn_	π_rep_	K_syn_	K_rep_	H_norm_	D
*AOS5*	26	1968	1445	47	0.00616	0.00663	0.00364	0.06208	0.01555	−0.8616	−0.8077
*CathL*	24	1879	1125	73	0.00862	0.01076	0.00388	0.04540	0.02002	−0.2085	−1.2994
*GHf10*	24	1035	594	51	0.01140	0.01182	0.00834	0.04419	0.02211	−0.0153	−1.0412
*NFXL1*	24	3454	3336	12	0.00121	0.00290	0.00076	0.03332	0.01024	−0.8345	1.0215
*PAL1*	24	3249	3142	87	0.00597	0.01015	0.00126	0.06916	0.00577	−1.3019	−0.0611
*PPO1*	54	1798	1689	77	0.01253	0.03523	0.00643	0.06994	0.03620	−0.6785	1.0923*
*PPO2*	26	1506	1506	45	0.00815	0.01928	0.00495	0.05017	0.02225	−2.63**	0.0683
*PPO3*	22	1381	1381	38	0.00785	0.02244	0.00364	0.07873	0.01735	0.2956	0.0515
Average of defense genes					0.00775	0.01473	0.00410	0.05524	0.01849		
Genome-wide average					0.00425	0.01202	0.00165	0.04758	0.00994	−0.5720	−0.4240

N, number of sampled haplotypes; Sites, length of gene regions surveyed; S, number of segregating sites; π_tot_, π_syn_, π_rep_, average pairwise diversity per site for total, synonymous, and replacement sites, respectively; K_syn_, K_rep_, divergence calculated from *P. trichocarpa* for synonymous, and replacement sites, respectively; H_norm_, Fay and Wu's normalized H; D, Tajima's D. Data for genome-wide average are from Ingvarsson [34].

*p = 0.05,

**p = 0.01.

Gene fragments were amplified from diploid genomic DNA using polymerase chain reaction (PCR) and directly sequenced on a Beckman Coulter CEQ 2000XL capillary sequencer at Umeå Plant Science Center. Sequences were verified manually and contigs were assembled using the computer program Sequencher v4.0. Regions with missing or low-quality data were trimmed from all sequences. Multiple sequence alignments were made using Clustal W [30] and adjusted manually using BioEdit (http://www.mbio.ncsu.edu/BioEdit/bioedit.html). Alignments were annotated on the basis of the corresponding gene from the *P. trichocarpa* genome sequence (available at http://www.phytozome.net/poplar.php).

### Digital northerns

The full-length coding regions for all eight genes were obtained from the annotated *P. trichocarpa* genome (version 2.0, http://www.phytozome.net/poplar). We then obtained all ESTs from the experiments of Sterky et al. [31] and and Ralph et al. [18] and an unpublished study from the Arborea project (http://www.arborea.ulaval.ca/) from GenBank or from PopulusDB (http://www.populus.db.umu.se/) (ESTs downloaded on 2010-10-05). We used these EST resources to obtain “digital northern” analysis of all genes. These digital northern analyses were performed to verify that we had selected genes from the *Populus* genome that do show wound induction.

Publicly available EST data sets can confound such digital expression analyses in a number of ways and several measures were taken to minimize these effects. First, ESTs are usually sequenced from only one end of the cDNA clone but in some cases EST clones are sequenced from both ends. To identify such cases, all clone IDs were retrieved and duplicate IDs were excluded from the analysis. Second, a relevant comparison of EST abundance is best made if ESTs originate from non-normalized cDNA libraries. Normalization will introduce a certain level of noise in the data set and will lead to an underestimation of mRNA abundance for highly expressed genes. However, it is unlikely that it should change the rank order of genes with respect to gene expression, and data from normalized data still provide semi-quantitative data on transcript abundance. We included data from normalized libraries but these have been labeled in subsequent analyses to aid in interpreting the data. The EST data sets used were further derived from a number of different species of *Populus*, but the low sequence divergence seen in coding regions between different species in the genus suggest that this will not influence the estimation of expression profiles from the EST libraries [32].

Full-length coding sequences were filtered with the XBLAST program to mask out repetitive elements. Expression profiles were then obtained for all genes BLASTN searches against each EST data set using stringent matching criteria. Alignments were required to show at least a 90% identity across 100 bp to be recorded as a match, to avoid over-estimating transcript abundance of genes belonging to small gene families. The number of BLASTN hits was used as a proxy for expression levels and normalized by the total number of ESTs in each data set, to enable comparisons between different treatments and/or tissues. Because wounding has been shown to affect the expression of most genes involved in both primary and secondary metabolism [33], we have not included a housekeeping gene as reference control which is normally done in comparative studies of gene expression.

### Population genetic analyses

Estimates of nucleotide polymorphism (segregating sites (S), nucleotide diversity (π)), between-species divergences, for synonymous and non-synonymous sites separately, and statistical tests of neutrality (Tajima's D, Fay & Wu's H and the McDonald-Kreitman test), were obtained using the computer program DnaSP v.4.50.2 (http://www.ub.es/dnasp/). For analyses requiring an outgroup, a single sequence of the homologous region in *P. trichcarpa* was used. To provide an estimate of the distribution of genome-wide levels of polymorphism we used data from 77 loci corresponding to 76 unique genes evenly distributed throughout the genome of *Populus*. These reference genes were chosen without prior knowledge of their function [see 34 for more details] and we refer to them as collectively as “reference genes” hereafter.

In order to evaluate significance statistics of Fay and Wu's H and Tajima's D, we performed coalescent simulations using Richard Hudson's ms program (available at http://home.uchicago.edu/~rhudson1/source/mksamples.html). For each summary statistic the null distribution under neutrality was obtained from 10^4^ simulations, conditioned on the original sample configuration and sequence length. The demographic history of *P. tremula* is characterized by a moderately strong bottleneck that occurred roughly 770 kya and to account for this, coalescent simulations were performed based on the bottleneck model described in Ingvarsson [34]. Briefly, for each simulation replicate, parameters for the bottleneck model were drawn from the posterior distribution of these parameters as described in Ingvarsson [34]. The corresponding summary statistic calculated from the observed data was then compared to the full distribution of the simulated data sets to determine where they fell in the range of simulated values.

Since LD extends only a few hundred bps in *P. tremula* [34], it is possible that any signals of natural selection only affect a small region around the target site of selection. We therefore also performed sliding window analyses for synonymous diversity, Tajima's *D*, Fay and Wu's *H* and the K_A_/K_S_ ratio to study how patterns of polymorphism, divergence and site frequency spectra (SFS) vary across genes. The sliding window analysis of diversity at synonymous sites was performed using a 75 bp window that was moved in 8 bp increments across the gene, while the rest of the analyses were performed using a 250 bp window moved in 25 bp increments. Smaller window sizes yielded too little polymorphism in many windows, while larger windows increase the risk of averaging over too many sites with independent evolutionary histories, thereby reducing the power to detect regions with enhanced genetic differentiation. We used coalescent simulation to determine whether specific window segments showed significant deviations from neutral expectations. We simulated the same number of alleles that were present in our sample and simulations were run conditional on the number of segregating sites observed in each window. We performed simulations without recombination as we are simulating only short gene-segments. Furthermore, ignoring recombination is conservative with respect to identifying outlier-values for Tajima's D and Fay and Wu's H. For the K_A_/K_S_ ratio we adopted a slightly different method to assess outlier windows. We calculated the 95% percentile from the distribution of K_A_/K_S_ from the 76 control loci and used this value as a cutoff limit to assess which window-regions that show elevated K_A_/K_S_ ratios. We regard the sliding window analyses as exploratory and as a way to identify regions worth studying in greater detail and we therefore do not correct for multiple testing in the analysis.

## Results

### Digital northerns

**Figure 1:**
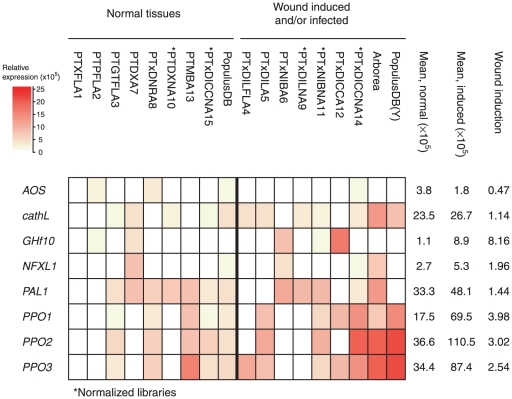
Heat map of digital northern analyses of expression data for eight defense-associated genes in *Populus*. Data sets were obtained from Ralph et al. (2006) (denoted by PT), Sterky et al (2004) (denoted by PopulusDB) or from unpublished data from the Arborea project (http://www.arborea.ulaval.ca/, denoted Arborea). Data sets obtained from normalized libraries are denoted by *. Wound induction gives the relative expression in data sets obtained from wounded or infected tissues compared to normal tissues.

We compared gene expression across different tissues using digital northerns based on available EST data sets (Figure 1). Although there is substantial variation across different tissues and wounding treatments, 7 of 8 genes studied here show a consistently higher induction in wounded or infected tissues (paired *t*-test, *t* = 3.07, *p* = 0.017, Figure 1). The only exception is *AOS5* which showed very low expression across all data sets we analyzed. This confirms that the majority of the genes we studied are indeed wound induced, showing higher gene expression following wounding.

### Intraspecific patterns of nucleotide polymorphism

Between 22 and 54 alleles were sequenced for *AOS5*, *CathL*, *GHf10*, *NFXL1*, *PAL1*, *PPO1*, *PPO2* and *PPO3* (Table 1). The complete coding region together with some flanking regions were sequenced for five of the genes (*AOS5*, *CathL*, *NFXL1*, *PAL1* and *PPO1*) while only a part of the coding regions was obtained for *GHf10*, *PPO2* and *PPO3*. Seven of the genes show substantial levels of polymorphism, with the number of segregating sites ranging from 38 to 87 (Table 1). *NFXL1*, by contrast, contained only 12 segregating sites despite having the longest contiguous stretch of sequence. Overall, the three *PPO* genes show higher diversity at both synonymous and non-synonymous sites, compared to the genome-wide average in *P. tremula*, while *AOS5*, *CathL*, and GHf10 are showing slightly to moderately reduced levels of sequence diversity at synonymous sites but elevated levels of diversity at non-synonymous. *NFXL1* and *PAL1* show relatively low levels of sequence diversity at both synonymous and non-synonymous sites (Table 1).

**Figure 2:**
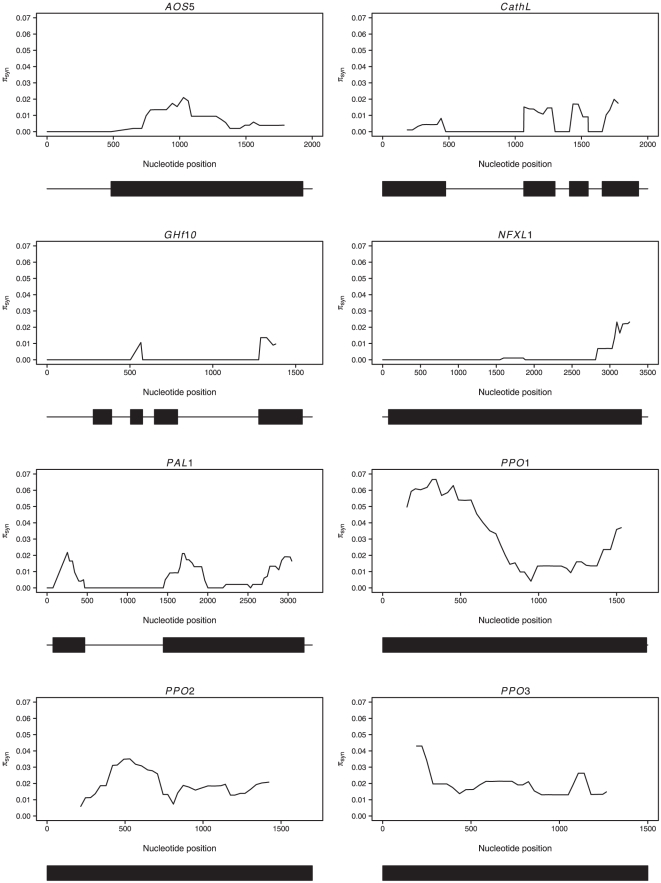
Sliding window of intraspecific diversity at synonymous sites (π_syn_). Values plotted are the midpoints of windows 75 bp wide at 8 bp increments. The corresponding gene structures are shown at the bottom of the plots: exons are indicated by boxes and non-coding regions (introns and UTRs) by lines.

The sliding window analysis of the synonymous diversity shows a striking dip in a region in the middle of *PPO1* with a three-fold reduction in polymorphism (Figure 2F). Even though *PPO2* shows a similar pattern, with a reduction of synonymous polymorphisms in the center of the gene, the reduction it is not as pronounced as in *PPO1*. The remaining six genes show a more homogenous distribution of polymorphism (Figure 2).

If any of the genes are under the influence of strong natural selection it should be possible to determine this from shifts in the site frequency spectrum (SFS). An excess of intermediate frequency variants in the SFS, as indicated by a positive Tajima's *D*, suggest that the gene might be under balancing selection whereas an excess of high frequency derived variants, indicated by a negative value of Fay and Wu's *H*, is a sign of directional selection, as high frequency derived variants are very rare under a neutral model. Two of the defense genes show significant shifts in the site frequency spectrum compared to neutral expectations (Table 1), with *PPO1* having a significantly positive Tajima's *D* (*D* = 1.09, p = 0.02) and *PPO2* have a significantly negative Fay and Wu's *H* (*H* = −2.63, p = 0.006). The other six genes (*AOS5, CathL, GHf10, NFXL1, PAL1 and PPO3*) do not show any deviations from neutral expectations (Table 1).

**Figure 3:**
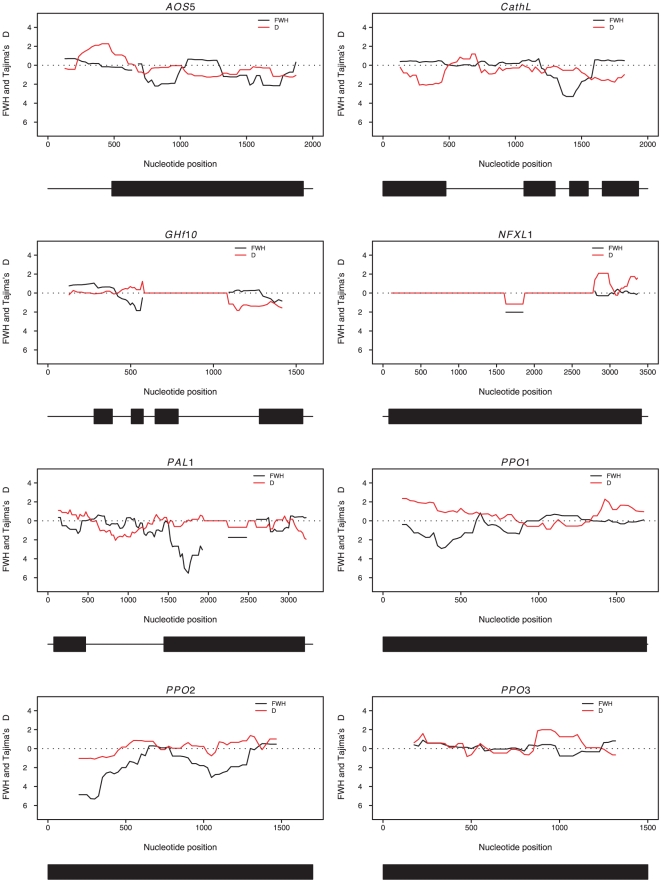
Sliding window of Tajima's D (red line) and Fay and Wu's normalized H (black line). Values plotted are the midpoints of windows 250 bp wide at 25 bp increments. Dotted line indicate strict neutrality. The corresponding gene structures are shown at the bottom of the plots: exons are indicated by boxes and non-coding regions (introns and UTRs) by lines.

The sliding window analyses show that in *PPO1*, a region at the 5′ end of the coding region harbors an excess of both intermediate- and high frequency variants (positive Tajima's D and negative Fay and Wu's H, Figure 3F). Furthermore, *PAL1* and *PPO2* show regions with very distinct negative values of Fay and Wu's H, for *PAL1* at the 5′ end of exon 2 and for *PPO2* most clearly at the 5′ end of the gene. For the remaining genes there are a few regions showing weak deviations from neutrality but none of the genes show any systematic patterns. The simulated confidence intervals for each window across the eight genes are presented in Table S1.

### Polymorphism and Divergence

Three of the defense genes (C*athL*, *GHf10* and *NFXL1*) have lower or equal divergence at synonymous sites compared to the genome-wide average, whereas the other five defense genes are showing a higher degree of divergence. At non-synonymous sites only *PAL1* and *NFXL1* exhibit a lower or equal degree of divergence compared to the genome-wide average (Table 1).

**Figure 4:**
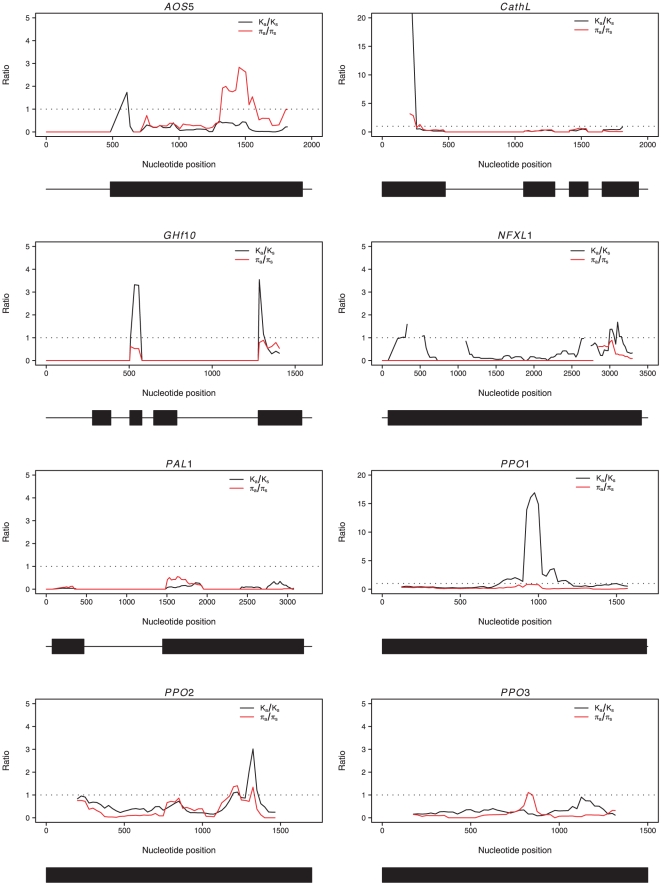
Sliding window of nonsynonymous-to-synonymous ratio of diversity (red line) and divergence (black line). Values plotted are the midpoints of windows 250 bp wide at 25 bp increments. Dotted line indicates the 95 percentile of the K_A_/K_S_ ratio for the 76 control loci (see text for further details) which is equal to 1.14.The corresponding gene structures are shown at the bottom of the plots: exons are indicated by boxes and non-coding regions (introns and UTRs) by lines.

None of the eight defense genes are showing K_A_/K_S_ ratios that exceed unity. However, sliding window analyses identifies a 600 bp region in the middle of the *PPO1* gene that show a greatly elevated K_A_/K_S_ ratio (K_A_/K_S_>15, Figure 4). C*athL*, *GHf10*, *PPO2*, and to lesser extent *AOS5* and *NFXL1*, are also harboring small regions with elevated K_A_/K_S_ ratios, but these are less distinct than the very clear peak of elevated K_A_/K_S_ seen in *PPO1*. These results suggest that adaptive evolution has occurred in the *PPO1* gene, as evidenced by the preferential fixation of non-synonymous mutations compared to synonymous mutations. In line with this, we find that a McDonald-Kreitman (MK) test, which compares the rate of divergence to levels of standing nucleotide polymorphism within species, also identifies *PPO1* as a gene whichs show a significant deviation from neutral expectations with a -log_10_ neutrality index (NI) of 0.721 (Fisher's exact test, p = 3×10^−5^), suggesting again preferential fixation of non-synonymous mutations at *PPO1*. None of the other seven genes show any deviations from expectations in the MK-tests (Table 2).

**Table 2:** Number of fixed and polymorphic mutations at synonymous and replacement sites in the coding regions of *Populus tremula* inducible defense genes.

	Synonymous		Replacement			
Locus	Fixed	Poly	Fixed	Poly	-log_10_(NI)	MK (*p* value)
*AOS*	16	11	11	19	−0.40	2.93 (0.09)
*CathL*	10	13	13	21	−0.09	0.16 (0.69)
*GHf10*	3	5	4	16	−0.38	0.89 (0.35)
*NFXL1*	23	6	24	6	0.02	0.004 (0.95)
*Pal1*	26	18	5	9	−0.41	2.35 (0.13)
*PPO1*	10	44	37	31	0.72	17.16 (3e-5)
*PPO2*	7	25	14	21	0.38	2.59 (0.11)
*PPO3*	19	21	14	18	−0.06	0.10 (0.75)

## Discussion

We have examined patterns of nucleotide variation in eight wound-induced genes from European aspen (*P. tremula*). These genes are representative of the two main pathways that control wound-induced defense responses, the octadecanoid and the salicylate pathway [35]. The digital northern analyses confirm that all but one gene show increased gene transcription following wounding, confirming that these genes (with the exception of *AOS5*) are wound induced (Figure 1), in line with a number of recent studies that have investigated transcriptional changes following wounding and herbivory in *Populus* [17]–[19], [36], [37].

With a few notable exceptions (see below), patterns of nucleotide polymorphism in the eight wound induced genes conform to neutral expectations, and most genes also show low K_A_/K_S_ ratios, indicating that purifying selection acted on most non-synonymous polymorphisms in these genes.

While the majority of genes we have studied show few sign of selection apart from a purifying selection on non-synonymous polymorphisms, two genes from the polyphenol oxidase gene family, *PPO1* and *PPO2*, show several signs that are indicative of the action of natural selection at these two genes. Both *PPO1* and *PPO2* show significant skew in their site frequency spectra, at least for parts of their coding regions. *PPO1* show an excess of intermediate frequency variants across the entire gene (*D* = 1.09, *p* = 0.02), and the sliding window analysis of the SFS shows that this patterns is most pronounced in the regions close to the 5′ and 3′ ends of the gene. In addition, the 5′ end of the gene shows a clear excess of high frequency derived variants (negative Fay and Wu's *H*, Figure 3). Furthermore, even though the K_A_/K_S_ ratio does not exceed unity for the entire *PPO1* gene, a 600 bp region in the center of the gene display a greatly elevated K_A_/K_S_ ratio, exceeding unity by up to twentyfold (Figure 4) and an MK-test also indicates that *PPO1* harbors an excess of non-synonymous fixations. In addition, the same region showing an elevated K_A_/K_S_ ratio also shows markedly reduced levels of synonymous polymorphism compared to surrounding regions in the *PPO1* gene (Figure 2). Taken together, this suggests that *PPO1* has experienced recurrent adaptive evolution, with a preferential fixation of non-synonymous mutations. The deviations we observe in the SFS, with an excess of both high frequency derived and intermediate frequency variants hints at a strong haplotype structure in the 5′ region of *PPO1* (Figure 3), that could be a signature of a recently completed sweep at *PPO1* (eg. [38]–[40]).

When searching for conserved domains in *PPO1* (www.ncbi.nlm.nih.gov/Structure/cdd/wrpsb.cgi), we found that the 600 bp region with elevated K_A_/K_S_ ratio and reduced synonymous diversity is located close to the end of the tyrosinase superfamily domain, the domain that contains the active site for all *PPOs*. This domain consists of a di-nuclear copper center, with two copper ions, each coordinated to three histidine side chains [41]. Both the peak in the K_A_/K_S_ ratio and the deepest dip of the intraspecific diversity lies within the second histidine residue of the tyrosinase domain, hinting at the possibility that the possible target of selection might affect the enzymatic catalytic properties of *PPO1*. Further biochemical characterizations of the different allelic variants of *PPO1* are required to test this hypothesis.

In contrast to *PPO1*, *PPO2* does not show an elevated K_A_/K_S_ ratio, with the exception of a short region in the 3′ end of the gene (Figure 4) in a domain that is generally thought to be involved in activation of the enzyme by proteolytic cleavage [41]. Furthermore, *PPO2* shows a significant excess of high frequency derived variants across the entire gene (Fay and Wu's *H* = −2.63, *p* = 0.006). The sliding window analysis also identified a region in the middle of the gene that shows no deviation from neutrality, but that is flanked on both sides by regions showing an excess of high frequency derived variants (Figure 3)- a sign that could indicate that also *PPO2* went through a recent selective sweep [38], [39]. The MK-test only indicated a weak trend towards an excess of non-synonymous fixations at *PPO2* (*p* = 0.107), but the MK-test only detects significant deviations from neutrality when the gene has been under long-term adaptive evolution. Interestingly, judging by the location of possible flanking regions showing significant deviations in the SFS, a putative target of selection in *PPO2* would also be located in the tyrosinase domain of the protein, just as in *PPO1*.

The two genes showing signs of either long-term or more recent natural selection are members of the polyphenol oxidase gene family, a small gene family consisting of up to 10–12 different members in the *Populus* genome [29]. PPO enzymes are generally categorized as generalist defenses when it comes to substrate specificity [29], as the function of PPOs in the defensive response is to catalyze the oxidation of diphenolic compounds into quinones, which in turn cross-link or alkylate essential amino acids and sulfhydryl groups in dietary proteins [29]. This leads to a general reduction in the nutritional value of the tissue, and ultimately to lower growth rates or even starving herbivores [29]. Transgenic plants with altered expression of PPOs have also been shown to have detrimental effects on insect performance [42]–[47], although results vary between different herbivore guilds and with the age of herbivores [48] In *Populus* both *PPO1* and *PPO2* have been shown to be wound-inducibe, with *PPO1* being exclusively expressed in damaged leaves while *PPO2* is primarily expressed in stems, petioles and roots [28].

Genes like *PPO*, involved in generalized defense, often show few signs of natural selection compared to genes involved in specialized defenses [5], [6] and it may therefore seem rather surprising that two of the three PPO genes included in this study show signs of natural selection. In fact, the two *PPO* genes in this study show patterns of selection that are reminiscent of the patterns found from trypsin inhibitor (TI) genes in *Populus* [20], [49], [50]. The TI genes are generally thought to represent a much more specialized defense mechanism than that provided by the *PPO* genes due to the highly selective nature of TI genes when targeting specific protases [11], [51]. However, recent results have shown that *PPO* proteins are not only resistant to proteolysis in insect guts, but that limited proteolysis actually is required to activate the protein from the latent form in which it is stored in plant leaves [27], [41], [45]. In this respect it is interesting to note that the region showing strong signs of adaptive evolution in *PPO1*, and to a lesser extent also in *PPO2*, is located close to the active site of the PPO protein and also close to a region that has been speculated to be involved in controlling conditions under which the PPO protein becomes activated in the insect gut [29], [41]. One possibility is therefore that the signs of selection we observe in *PPO1* and *PPO2* are linked to natural selection in herbivores, preventing or reducing the activation of the PPO protein, and to counter-selection in plants for maintaining this function. Such a co-evolutionary scenario is clearly a speculation at this point and more data on both the functional consequences of mutations at sites targeted by natural selection and possible counter-selection in herbivores are needed before this question can be answered conclusively.

To conclude, we have shown that patterns of polymorphism and divergence at most of the eight wound-induced genes we studied showed little deviation from neutrality. The exceptions a two polyphenol oxidase genes which both appear to have been the target of recent selective sweeps, and in the case of *PPO1*, also long-term adaptive evolution. Our results support the notion that the majority of wound-induced genes are not under strong selection in *Populus*. However, the results show that some biochemical herbivore defenses, that are strongly induced following mechanical damage or insect attack, show signs of adaptive evolution in *Populus* [49], [50]. These results supports data from other species, such as Arabidopsis and maize, which have found that the the majority of defense-associated genes show few signs of selection and that genes showing signs of natural selection are usually involved in specialist defenses [5], [6], [52], [53].

## Supporting Information

Table S1 Number of segregating sites, nucleotide diversity, observed values of Tajima's *D* and Fay and Wu's *H* and their simulated 95% confidence intervals for the sliding window analysis.
(PDF)
