# Supplementary material for: Molecular Population Genetics of Elicitor-Induced Resistance Genes in European Aspen (Populus tremula L., Salicaceae)
Source: PLoS One. 2011 Sep 19;6(9):e24867. doi: 10.1371/journal.pone.0024867 (PMC3176232; doi:10.1371/journal.pone.0024867)
Supplement: Table S1 — Number of segregating sites, nucleotide diversity, observed values of Tajima's D and Fay and Wu's H and their simulated 95% confidence intervals for the sliding window analysis. (PDF) [file pone.0024867.s001.pdf]

AOS5

Midpoint	S	D observed	Simulated D (0.025)	Simulated D (0.975)	H observed	Simulated H (0.025)	Simulated H (0.975)
129	6	-0.336	-1.692	1.813	0.694	-2.458	0.987
154	8	-0.426	-1.677	1.913	0.700	-2.203	0.968
179	8	-0.426	-1.783	1.812	0.700	-2.662	0.992
204	8	-0.426	-1.700	1.752	0.700	-2.585	0.991
232	13	0.919	-1.733	1.799	0.572	-2.439	1.010
257	12	1.269	-1.744	1.855	0.402	-2.263	1.004
283	11	1.498	-1.632	1.863	0.349	-2.367	1.035
308	9	1.692	-1.743	1.834	0.139	-2.909	0.998
333	10	1.961	-1.770	1.776	0.197	-2.234	0.991
358	11	2.108	-1.642	1.742	0.190	-2.222	0.973
383	11	2.108	-1.635	1.870	0.190	-2.343	1.022
408	11	2.267	-1.684	1.810	-0.159	-2.368	0.998
433	11	2.267	-1.702	1.736	-0.159	-2.452	1.022
458	11	2.267	-1.784	1.792	-0.159	-2.868	0.998
483	6	1.436	-1.691	2.028	-0.193	-2.670	0.967
508	5	1.092	-1.696	1.899	-0.206	-2.568	0.948
533	5	1.092	-1.696	2.018	-0.206	-2.447	0.926
558	5	1.092	-1.709	1.957	-0.206	-2.492	0.959
583	3	0.908	-1.734	1.954	-0.450	-2.165	0.887
608	2	0.134	-1.513	2.008	-0.511	-2.809	0.823
633	2	0.134	-1.513	2.008	-0.511	-2.789	0.803
658	0	0.000	NA	NA	NA	NA	NA
683	1	-0.714	-1.156	1.591	0.150	-2.987	0.631
708	1	-0.714	-1.156	1.591	0.150	-3.456	0.631
733	2	-0.935	-1.513	1.946	-1.344	-2.428	0.823
758	5	-0.758	-1.709	1.878	-1.820	-2.502	0.970
783	6	-0.319	-1.703	1.698	-1.551	-2.534	0.948
808	7	-0.268	-1.722	1.874	-2.187	-2.506	1.010
833	7	-0.268	-1.722	1.935	-2.187	-2.281	0.984
858	8	-0.228	-1.650	1.785	-1.955	-2.577	0.968
883	8	-0.228	-1.709	1.854	-1.955	-2.248	0.960
908	8	-0.228	-1.728	1.830	-1.955	-2.819	0.999
933	8	-0.043	-1.709	1.960	-1.906	-2.367	0.992
958	8	-0.043	-1.672	1.900	-1.906	-2.592	1.007
983	8	-0.043	-1.677	1.923	-0.991	-2.311	0.976
1008	6	-0.041	-1.692	2.022	-0.247	-2.496	0.987
1033	6	-0.597	-1.825	1.964	-0.231	-2.931	0.938
1058	8	-0.925	-1.700	1.807	0.645	-2.584	1.007
1083	8	-0.925	-1.806	1.891	0.645	-2.538	0.976
1108	8	-1.109	-1.691	1.863	0.596	-2.312	0.999
1133	8	-1.109	-1.673	1.776	0.596	-2.461	0.952
1164	8	-1.109	-1.709	1.808	0.596	-2.234	1.007
1201	7	-1.249	-1.701	1.822	0.496	-2.419	0.976
1226	7	-1.249	-1.711	1.945	0.496	-2.332	1.002
1251	6	-1.193	-1.703	1.877	0.525	-2.401	0.938
1276	6	-1.193	-1.825	1.842	0.525	-2.554	0.929
1301	8	-1.109	-1.747	1.776	-0.215	-2.460	0.991
1326	6	-0.828	-1.691	1.779	-1.188	-2.525	0.948

1351	6	-0.828	-1.825	1.977	-1.188	-2.450	0.967
1376	6	-0.962	-1.703	1.906	-1.227	-2.389	0.958
1401	6	-0.962	-1.703	1.924	-1.227	-2.883	0.948
1426	6	-0.962	-1.704	1.912	-1.227	-2.467	0.948
1451	7	-0.468	-1.711	1.879	-1.050	-2.280	0.976
1476	7	-0.468	-1.819	1.806	-1.050	-2.461	0.984
1501	7	-0.468	-1.722	1.766	-1.050	-2.620	0.993
1526	7	-0.468	-1.808	1.874	-2.092	-2.331	0.976
1551	4	-0.154	-1.707	1.948	-1.868	-2.808	0.913
1576	3	-0.216	-1.734	1.954	-1.086	-2.381	0.887
1601	3	-0.216	-1.734	2.100	-1.086	-2.304	0.887
1626	4	-0.288	-1.707	1.987	-2.117	-2.808	0.952
1651	4	-0.288	-1.707	1.995	-2.117	-3.009	0.913
1676	4	-0.288	-1.707	1.995	-2.117	-2.688	0.874
1701	4	-1.166	-1.546	1.994	-2.153	-2.573	0.913
1726	4	-1.166	-1.707	2.034	-2.153	-2.691	0.926
1751	4	-1.166	-1.887	1.917	-2.153	-2.933	0.913
1776	3	-1.071	-1.734	1.955	-1.076	-2.288	0.871
1801	4	-1.166	-1.707	1.814	-0.881	-2.689	0.887
1826	5	-1.237	-1.709	1.885	-0.725	-2.183	0.915
1851	5	-1.237	-1.709	1.957	-0.725	-2.922	0.937
1873	3	-1.071	-1.734	1.954	0.323	-2.460	0.887

cathL

Midpoint	S	D observed	Simulated D (0.025)	Simulated D (0.975)	H observed	Simulated H (0.025)	Simulated H (0.975)
130	6	-0.221	-1.654	1.968	0.393	-2.349	0.963
155	5	-0.804	-1.831	1.831	0.420	-2.805	0.937
180	5	-0.804	-1.667	1.784	0.420	-2.767	0.950
205	7	-1.235	-1.773	1.907	0.465	-2.926	0.995
225	6	-1.047	-1.667	1.988	0.443	-2.750	0.969
250	6	-1.047	-1.667	1.873	0.443	-2.219	0.935
275	6	-2.083	-1.697	1.771	0.298	-2.687	0.969
300	7	-2.026	-1.718	1.962	0.367	-2.373	0.995
325	8	-2.096	-1.651	1.747	0.394	-2.490	0.990
350	7	-2.026	-1.646	1.877	0.367	-2.503	0.980
375	7	-2.026	-1.658	1.816	0.367	-2.383	0.980
399	7	-1.899	-1.719	1.738	0.405	-2.865	0.990
418	9	-1.947	-1.666	2.078	0.488	-2.403	1.004
443	7	-1.772	-1.646	1.895	0.444	-2.183	0.990
468	10	-0.560	-1.739	1.850	0.100	-2.544	1.016
493	12	-0.007	-1.806	1.781	0.031	-2.407	1.023
518	11	0.204	-1.779	1.892	-0.007	-2.187	1.012
557	10	0.356	-1.653	1.878	-0.082	-2.491	1.004
582	10	0.862	-1.716	1.973	-0.022	-2.238	1.008
607	11	0.671	-1.780	1.804	0.050	-2.651	1.023
632	11	0.671	-1.679	1.625	0.050	-2.325	1.005
656	11	0.671	-1.679	1.920	0.050	-2.156	0.998
671	9	1.192	-1.760	1.843	-0.066	-2.293	1.016
696	9	1.192	-1.750	1.768	-0.066	-2.390	1.004
721	7	0.131	-1.575	1.931	0.303	-2.059	0.990
744	5	-0.789	-1.682	1.894	0.429	-2.555	0.957
768	4	-0.459	-1.690	1.974	0.406	-2.532	0.922
800	8	-0.913	-1.673	1.747	0.012	-2.450	1.000
823	8	-0.913	-1.749	1.882	0.012	-2.213	1.000
848	7	-0.836	-1.809	1.810	-0.073	-2.700	1.010
879	11	-0.217	-1.841	1.821	0.276	-2.216	1.002
904	13	-0.555	-1.762	1.756	0.331	-2.126	1.050
931	17	-0.344	-1.742	1.700	0.167	-2.238	1.019
956	17	-0.344	-1.634	1.728	0.167	-2.211	1.036
981	17	-0.284	-1.697	1.737	0.194	-2.611	1.026
1004	17	-0.284	-1.757	1.683	0.194	-2.587	1.051
1028	17	-0.284	-1.665	1.768	0.194	-2.547	1.039
1052	13	0.069	-1.708	1.846	0.468	-2.407	1.026
1075	13	-0.129	-1.801	1.835	0.485	-2.399	1.044
1101	15	-0.257	-1.772	1.803	0.593	-2.530	1.045
1126	12	-0.688	-1.671	1.815	0.418	-2.474	1.019
1151	10	-0.181	-1.680	1.697	0.413	-2.398	1.023
1176	6	-0.501	-1.667	1.906	0.696	-2.168	0.963
1198	8	-0.837	-1.651	1.996	-0.321	-2.385	1.000
1216	7	-0.751	-1.592	1.757	-0.422	-2.735	0.980
1240	10	-0.226	-1.677	1.729	-1.053	-2.145	1.027
1265	10	-0.226	-1.738	1.778	-1.053	-2.207	1.020
1290	12	-0.089	-1.687	1.900	-1.327	-2.306	0.999

1315	12	-0.154	-1.713	1.850	-1.345	-2.188	1.023
1359	13	-0.534	-1.650	1.807	-3.101	-2.234	1.023
1386	12	-0.530	-1.725	1.834	-3.298	-2.336	0.983
1411	12	-0.530	-1.729	1.762	-3.298	-2.596	1.023
1436	12	-0.530	-1.721	1.826	-3.298	-2.221	1.049
1458	10	-0.253	-1.738	1.842	-2.682	-2.570	1.020
1476	12	-0.390	-1.803	1.772	-2.344	-2.606	1.052
1501	10	-0.763	-1.824	1.684	-1.718	-2.459	1.004
1525	11	-0.917	-1.766	1.837	-1.615	-2.366	0.987
1550	9	-1.218	-1.681	1.872	-1.357	-2.219	0.979
1575	10	-1.526	-1.725	1.779	-1.320	-2.422	1.001
1600	8	-1.282	-1.754	1.817	0.568	-2.458	0.968
1625	9	-1.415	-1.667	1.768	0.585	-2.556	1.000
1650	8	-1.591	-1.640	1.920	0.483	-2.208	0.986
1675	8	-1.591	-1.673	1.877	0.483	-2.418	0.990
1700	9	-1.499	-1.666	1.881	0.566	-2.290	0.988
1725	8	-1.684	-1.691	1.892	0.463	-2.637	0.981
1750	8	-1.776	-1.754	1.914	0.442	-2.413	0.990
1775	9	-1.312	-1.642	1.734	0.527	-2.444	0.975
1800	9	-1.312	-1.696	2.069	0.527	-2.178	0.991
1823	7	-0.993	-1.772	1.727	0.486	-2.414	0.995

GHf10

Midpoint	S	D observed	Simulated D (0.025)	Simulated D (0.975)	H observed	Simulated H (0.025)	Simulated H (0.975)
128	13	-0.180	-1.689	1.770	0.759	-2.278	0.979
153	14	0.272	-1.739	1.752	0.853	-2.353	1.014
178	15	0.100	-1.746	1.818	0.870	-2.178	1.051
203	15	0.100	-1.676	1.726	0.870	-2.113	1.012
223	16	0.004	-1.694	1.745	0.908	-2.390	1.057
253	15	0.014	-1.624	1.902	0.974	-2.302	1.040
278	17	-0.084	-1.662	1.849	1.049	-2.204	1.002
303	16	-0.050	-1.650	1.685	0.531	-2.242	1.039
328	15	-0.002	-1.699	1.837	0.630	-2.592	1.012
353	16	0.196	-1.658	1.919	0.647	-2.360	1.000
373	16	0.196	-1.727	1.606	0.647	-2.346	1.024
398	14	-0.101	-1.702	1.698	0.503	-2.361	1.026
423	13	-0.006	-1.783	1.770	-0.351	-2.200	1.041
448	13	0.350	-1.639	1.737	-0.602	-2.478	1.004
469	12	0.481	-1.709	1.854	-0.691	-2.133	1.023
481	11	0.434	-1.746	1.854	-0.841	-2.318	1.005
498	8	0.689	-1.754	1.828	-1.254	-2.217	0.990
510	6	0.556	-1.694	1.907	-1.043	-2.193	0.963
523	4	0.586	-1.884	1.771	-1.318	-2.892	0.884
535	2	0.360	-1.515	2.056	-1.849	-2.726	0.819
546	2	0.360	-1.515	1.966	-1.849	-2.726	0.819
558	2	0.360	-1.515	1.966	-1.849	-2.831	0.796
571	1	1.232	-1.159	1.573	-0.510	-3.314	0.631
583	0	0.000	NA	NA	NA	NA	NA
601	0	0.000	NA	NA	NA	NA	NA
626	0	0.000	NA	NA	NA	NA	NA
651	0	0.000	NA	NA	NA	NA	NA
676	0	0.000	NA	NA	NA	NA	NA
701	0	0.000	NA	NA	NA	NA	NA
726	0	0.000	NA	NA	NA	NA	NA
751	0	0.000	NA	NA	NA	NA	NA
776	0	0.000	NA	NA	NA	NA	NA
801	0	0.000	NA	NA	NA	NA	NA
1069	0	0.000	NA	NA	NA	NA	NA
1081	0	0.000	NA	NA	NA	NA	NA
1094	1	-1.159	-1.159	1.573	0.088	-3.314	0.631
1106	1	-1.159	-1.159	1.573	0.088	-3.328	0.631
1118	1	-1.159	-1.159	1.573	0.088	-3.858	0.631
1130	3	-1.494	-1.733	1.920	0.241	-2.312	0.880
1143	5	-1.831	-1.667	1.839	0.309	-1.851	0.976
1155	5	-1.831	-1.667	1.839	0.309	-2.439	0.924
1170	8	-1.412	-1.706	1.801	0.126	-2.836	1.013
1182	9	-1.253	-1.681	1.813	0.244	-2.502	0.992
1201	9	-1.253	-1.740	1.808	0.244	-2.535	0.987
1226	10	-1.377	-1.784	2.054	0.275	-2.528	1.031
1251	10	-1.377	-1.684	1.774	0.275	-2.269	0.989
1276	11	-1.396	-1.771	1.816	0.337	-2.311	0.998
1300	12	-1.338	-1.609	2.081	-0.328	-2.037	0.996

1325	10	-1.197	-1.698	1.756	-0.469	-2.391	1.023
1346	8	-0.886	-1.640	1.887	-0.600	-2.381	0.991
1369	12	-1.102	-1.656	1.809	-0.989	-2.316	1.016
1394	12	-1.427	-1.752	1.715	-0.698	-2.873	1.016
1414	11	-1.562	-1.746	1.762	-0.838	-2.663	1.002

NFXL1

Midpoint	S	D observed	Simulated D (0.025)	Simulated D (0.975)	H observed	Simulated H (0.025)	Simulated H (0.975)
113	0	0.000	NA	NA	NA	NA	NA
138	0	0.000	NA	NA	NA	NA	NA
163	0	0.000	NA	NA	NA	NA	NA
188	0	0.000	NA	NA	NA	NA	NA
237	0	0.000	NA	NA	NA	NA	NA
262	0	0.000	NA	NA	NA	NA	NA
287	0	0.000	NA	NA	NA	NA	NA
312	0	0.000	NA	NA	NA	NA	NA
337	0	0.000	NA	NA	NA	NA	NA
355	0	0.000	NA	NA	NA	NA	NA
375	0	0.000	NA	NA	NA	NA	NA
400	0	0.000	NA	NA	NA	NA	NA
425	0	0.000	NA	NA	NA	NA	NA
450	0	0.000	NA	NA	NA	NA	NA
475	0	0.000	NA	NA	NA	NA	NA
500	0	0.000	NA	NA	NA	NA	NA
525	0	0.000	NA	NA	NA	NA	NA
550	0	0.000	NA	NA	NA	NA	NA
575	0	0.000	NA	NA	NA	NA	NA
600	0	0.000	NA	NA	NA	NA	NA
625	0	0.000	NA	NA	NA	NA	NA
650	0	0.000	NA	NA	NA	NA	NA
675	0	0.000	NA	NA	NA	NA	NA
700	0	0.000	NA	NA	NA	NA	NA
725	0	0.000	NA	NA	NA	NA	NA
750	0	0.000	NA	NA	NA	NA	NA
775	0	0.000	NA	NA	NA	NA	NA
800	0	0.000	NA	NA	NA	NA	NA
825	0	0.000	NA	NA	NA	NA	NA
850	0	0.000	NA	NA	NA	NA	NA
875	0	0.000	NA	NA	NA	NA	NA
900	0	0.000	NA	NA	NA	NA	NA
925	0	0.000	NA	NA	NA	NA	NA
950	0	0.000	NA	NA	NA	NA	NA
975	0	0.000	NA	NA	NA	NA	NA
1000	0	0.000	NA	NA	NA	NA	NA
1025	0	0.000	NA	NA	NA	NA	NA
1050	0	0.000	NA	NA	NA	NA	NA
1075	0	0.000	NA	NA	NA	NA	NA
1100	0	0.000	NA	NA	NA	NA	NA
1125	0	0.000	NA	NA	NA	NA	NA
1150	0	0.000	NA	NA	NA	NA	NA
1175	0	0.000	NA	NA	NA	NA	NA
1200	0	0.000	NA	NA	NA	NA	NA
1225	0	0.000	NA	NA	NA	NA	NA
1250	0	0.000	NA	NA	NA	NA	NA
1275	0	0.000	NA	NA	NA	NA	NA
1300	0	0.000	NA	NA	NA	NA	NA

1325	0	0.000	NA	NA	NA	NA	NA
1350	0	0.000	NA	NA	NA	NA	NA
1375	0	0.000	NA	NA	NA	NA	NA
1400	0	0.000	NA	NA	NA	NA	NA
1425	0	0.000	NA	NA	NA	NA	NA
1450	0	0.000	NA	NA	NA	NA	NA
1475	0	0.000	NA	NA	NA	NA	NA
1500	0	0.000	NA	NA	NA	NA	NA
1525	0	0.000	NA	NA	NA	NA	NA
1550	0	0.000	NA	NA	NA	NA	NA
1575	0	0.000	NA	NA	NA	NA	NA
1600	0	0.000	NA	NA	NA	NA	NA
1625	1	-1.159	-1.159	1.574	-2.017	-3.314	0.631
1650	1	-1.159	-1.159	1.596	-2.017	-3.314	0.631
1675	1	-1.159	-1.159	1.596	-2.017	-3.314	0.631
1700	1	-1.159	-1.159	1.573	-2.017	-3.858	0.631
1725	1	-1.159	-1.159	1.573	-2.017	-3.314	0.631
1750	1	-1.159	-1.159	1.573	-2.017	-3.314	0.631
1775	1	-1.159	-1.159	1.573	-2.017	-2.806	0.631
1800	1	-1.159	-1.159	1.573	-2.017	-3.314	0.631
1825	1	-1.159	-1.159	1.573	-2.017	-3.314	0.631
1850	1	-1.159	-1.159	1.574	-2.017	-3.328	0.631
1875	0	0.000	NA	NA	NA	NA	NA
1900	0	0.000	NA	NA	NA	NA	NA
1925	0	0.000	NA	NA	NA	NA	NA
1950	0	0.000	NA	NA	NA	NA	NA
1975	0	0.000	NA	NA	NA	NA	NA
2000	0	0.000	NA	NA	NA	NA	NA
2025	0	0.000	NA	NA	NA	NA	NA
2050	0	0.000	NA	NA	NA	NA	NA
2075	0	0.000	NA	NA	NA	NA	NA
2100	0	0.000	NA	NA	NA	NA	NA
2125	0	0.000	NA	NA	NA	NA	NA
2150	0	0.000	NA	NA	NA	NA	NA
2175	0	0.000	NA	NA	NA	NA	NA
2200	0	0.000	NA	NA	NA	NA	NA
2225	0	0.000	NA	NA	NA	NA	NA
2250	0	0.000	NA	NA	NA	NA	NA
2275	0	0.000	NA	NA	NA	NA	NA
2300	0	0.000	NA	NA	NA	NA	NA
2325	0	0.000	NA	NA	NA	NA	NA
2350	0	0.000	NA	NA	NA	NA	NA
2375	0	0.000	NA	NA	NA	NA	NA
2400	0	0.000	NA	NA	NA	NA	NA
2425	0	0.000	NA	NA	NA	NA	NA
2450	0	0.000	NA	NA	NA	NA	NA
2475	0	0.000	NA	NA	NA	NA	NA
2500	0	0.000	NA	NA	NA	NA	NA
2525	0	0.000	NA	NA	NA	NA	NA
2550	0	0.000	NA	NA	NA	NA	NA
2575	0	0.000	NA	NA	NA	NA	NA
2600	0	0.000	NA	NA	NA	NA	NA

2625	0	0.000	NA	NA	NA	NA	NA
2650	0	0.000	NA	NA	NA	NA	NA
2675	0	0.000	NA	NA	NA	NA	NA
2700	0	0.000	NA	NA	NA	NA	NA
2725	0	0.000	NA	NA	NA	NA	NA
2750	0	0.000	NA	NA	NA	NA	NA
2775	0	0.000	NA	NA	NA	NA	NA
2800	1	1.391	-1.159	1.596	0.215	-3.328	0.631
2825	2	1.714	-1.515	1.966	-0.241	-2.445	0.819
2850	3	2.090	-1.733	2.079	-0.287	-2.231	0.871
2875	3	2.090	-1.733	1.841	-0.287	-2.612	0.880
2900	3	2.090	-1.733	1.899	-0.287	-2.259	0.880
2925	3	2.090	-1.733	1.977	-0.287	-2.309	0.880
2950	3	2.090	-1.733	2.079	-0.287	-2.095	0.880
2975	3	2.090	-1.733	2.057	-0.287	-2.095	0.871
3000	5	0.960	-1.682	1.768	-0.058	-2.465	0.944
3025	6	0.631	-1.810	1.770	0.036	-2.284	0.963
3050	5	0.247	-1.682	1.831	-0.092	-2.395	0.937
3075	4	-0.209	-1.690	1.965	0.234	-2.835	0.936
3100	4	-0.237	-1.884	2.002	0.406	-2.998	0.929
3125	6	0.167	-1.797	1.915	0.271	-2.288	0.952
3150	7	0.517	-1.811	1.919	0.064	-2.268	0.980
3175	7	0.517	-1.809	1.774	0.064	-2.628	0.960
3200	8	0.709	-1.570	1.784	0.203	-2.219	1.022
3225	8	0.709	-1.749	1.899	0.203	-2.472	1.004
3250	6	1.313	-1.797	1.880	0.054	-2.461	0.969
3275	5	1.744	-1.831	1.784	-0.039	-2.729	0.918
3300	5	1.744	-1.686	1.831	-0.039	-2.497	0.944
3325	5	1.744	-1.682	1.902	-0.039	-2.298	0.918
3350	4	1.447	-1.694	2.011	-0.146	-2.345	0.944
3364	2	1.580	-1.515	1.966	-0.065	-2.587	0.796

PAL1

Midpoint	S	D observed	Simulated D (0.025)	Simulated D (0.975)	H observed	Simulated H (0.025)	Simulated H (0.975)
131	5	1.101	-1.682	1.972	0.362	-2.594	0.957
154	5	1.101	-1.667	1.933	0.362	-2.805	0.924
177	6	0.884	-1.668	1.941	-0.515	-2.484	0.952
202	6	0.884	-1.797	1.886	-0.515	-2.535	0.997
227	6	0.884	-1.654	1.989	-0.515	-2.377	0.974
250	5	0.749	-1.682	1.870	-0.723	-2.568	0.944
275	4	1.160	-1.690	1.900	-0.886	-2.427	0.929
300	3	0.638	-1.733	2.024	-0.900	-2.612	0.880
325	3	0.638	-1.733	2.079	-0.900	-2.453	0.871
350	3	0.638	-1.733	2.079	-0.900	-2.612	0.880
375	3	0.151	-1.733	2.079	-1.336	-2.095	0.871
400	4	0.688	-1.694	1.891	-1.073	-2.728	0.892
425	4	0.512	-1.884	1.900	0.016	-2.997	0.884
450	4	0.512	-1.690	1.983	0.016	-2.413	0.914
473	5	0.952	-1.682	1.839	0.014	-2.612	0.944
498	6	0.481	-1.797	1.818	0.063	-2.467	0.974
524	7	0.246	-1.684	1.853	0.145	-2.388	0.980
548	8	-0.056	-1.705	1.947	0.183	-2.617	0.977
573	8	-0.056	-1.868	1.914	0.183	-2.940	0.995
601	10	0.022	-1.784	1.847	0.372	-2.445	1.012
626	10	-0.547	-1.699	1.747	0.617	-2.421	1.001
651	10	-1.052	-1.680	1.923	0.558	-2.391	1.027
678	9	-1.001	-1.707	1.709	0.504	-2.572	1.004
703	9	-1.001	-1.770	1.793	0.504	-2.605	1.004
726	10	-1.201	-1.729	1.752	-0.338	-2.356	0.993
751	9	-1.060	-1.697	1.932	-0.395	-2.091	1.000
772	8	-1.005	-1.684	1.719	-0.495	-2.381	0.972
793	12	-1.504	-1.598	1.702	-0.308	-2.078	0.986
817	12	-1.504	-1.776	1.733	-0.308	-2.346	1.029
842	14	-2.058	-1.764	1.934	-0.324	-2.496	1.000
867	13	-1.751	-1.823	1.984	-0.291	-2.345	1.016
899	14	-1.621	-1.730	1.624	-0.801	-2.498	1.037
930	15	-1.688	-1.673	1.725	-0.744	-2.375	1.056
956	19	-1.427	-1.717	1.718	-0.408	-2.573	0.985
981	19	-1.644	-1.709	1.629	0.311	-2.426	1.036
1006	20	-1.695	-1.693	1.674	0.335	-2.506	1.023
1027	20	-1.695	-1.691	1.672	0.335	-2.419	1.032
1051	17	-1.178	-1.671	1.743	0.114	-2.631	1.029
1078	20	-1.287	-1.653	1.689	0.248	-2.285	1.060
1103	18	-1.145	-1.726	1.689	-0.575	-2.482	1.046
1128	19	-0.901	-1.675	1.707	-0.744	-2.041	1.021
1153	19	-0.765	-1.647	1.767	-0.211	-2.103	1.014
1175	18	-0.670	-1.810	1.830	-0.248	-2.435	1.017
1199	15	-0.866	-1.720	1.834	-1.359	-2.037	1.015
1223	13	-0.700	-1.772	1.763	-1.546	-2.506	1.035
1248	15	-0.188	-1.663	1.713	-1.217	-2.374	1.004
1273	16	-0.324	-1.731	1.712	-1.150	-2.308	1.049
1302	16	-0.354	-1.730	1.721	-1.261	-2.800	1.029

1326	15	0.259	-1.727	1.841	-1.696	-2.414	1.040
1351	13	0.661	-1.664	1.756	-0.990	-2.237	1.010
1376	11	0.350	-1.754	1.867	-0.956	-2.570	1.002
1401	10	0.103	-1.698	1.769	-1.094	-2.376	1.035
1424	10	0.103	-1.750	1.784	-1.094	-2.448	1.023
1452	9	0.364	-1.681	1.842	-0.159	-2.286	0.996
1477	10	0.361	-1.730	1.752	-0.033	-2.478	0.989
1502	8	-0.349	-1.663	1.795	-0.345	-2.776	1.018
1527	10	0.054	-1.698	1.814	-2.072	-2.322	1.016
1552	9	-0.060	-1.760	1.719	-2.725	-2.598	1.000
1575	7	-0.606	-1.784	1.756	-2.676	-2.423	0.990
1600	7	-0.606	-1.657	1.919	-2.676	-2.342	0.985
1625	7	-0.606	-1.682	1.895	-2.676	-2.362	0.975
1650	7	-0.606	-1.658	1.937	-3.802	-2.181	0.990
1675	8	-0.430	-1.684	1.779	-3.473	-2.517	0.981
1700	12	-0.096	-1.772	1.768	-4.985	-2.458	1.016
1725	11	-0.129	-1.641	1.871	-5.278	-2.662	1.044
1750	10	0.090	-1.824	1.833	-5.524	-2.384	0.978
1775	7	-0.032	-1.657	1.961	-4.370	-2.666	0.995
1800	6	0.167	-1.797	2.050	-3.632	-2.484	0.963
1825	6	0.167	-1.810	1.853	-3.632	-2.551	0.946
1850	6	0.167	-1.694	1.887	-3.632	-2.433	0.963
1875	6	0.167	-1.694	1.879	-3.632	-2.284	0.974
1900	5	0.592	-1.831	1.768	-2.657	-2.363	0.931
1925	4	0.503	-1.690	1.909	-3.053	-2.728	0.914
1950	0	0.000	NA	NA	NA	NA	NA
1975	0	0.000	NA	NA	NA	NA	NA
2000	0	0.000	NA	NA	NA	NA	NA
2025	0	0.000	NA	NA	NA	NA	NA
2050	0	0.000	NA	NA	NA	NA	NA
2075	0	0.000	NA	NA	NA	NA	NA
2100	0	0.000	NA	NA	NA	NA	NA
2125	0	0.000	NA	NA	NA	NA	NA
2150	0	0.000	NA	NA	NA	NA	NA
2175	0	0.000	NA	NA	NA	NA	NA
2200	0	0.000	NA	NA	NA	NA	NA
2225	0	0.000	NA	NA	NA	NA	NA
2250	1	-0.681	-1.159	1.573	-1.754	-3.314	0.631
2275	1	-0.681	-1.159	1.573	-1.754	-3.858	0.631
2300	1	-0.681	-1.159	1.596	-1.754	-3.314	0.631
2325	1	-0.681	-1.159	1.596	-1.754	-3.314	0.631
2350	1	-0.681	-1.159	1.573	-1.754	-3.314	0.631
2375	1	-0.681	-1.159	1.573	-1.754	-3.314	0.631
2400	1	-0.681	-1.159	1.573	-1.754	-3.314	0.631
2425	1	-0.681	-1.159	1.596	-1.754	-3.314	0.631
2450	1	-0.681	-1.159	1.573	-1.754	-3.858	0.631
2475	1	-0.681	-1.159	1.573	-1.754	-3.314	0.631
2500	0	0.000	NA	NA	NA	NA	NA
2525	0	0.000	NA	NA	NA	NA	NA
2550	0	0.000	NA	NA	NA	NA	NA
2575	0	0.000	NA	NA	NA	NA	NA
2600	1	-0.681	-1.159	1.573	0.159	-3.314	0.631

2625	1	-0.681	-1.159	1.573	0.159	-3.314	0.631
2650	1	-0.681	-1.159	1.573	0.159	-3.314	0.631
2675	1	-0.681	-1.159	1.573	0.159	-2.806	0.631
2700	1	-0.681	-1.159	1.573	0.159	-2.818	0.631
2725	1	-0.681	-1.159	1.573	0.159	-3.314	0.631
2750	2	-0.354	-1.515	1.966	0.339	-2.445	0.796
2775	3	-0.848	-1.733	2.079	-1.152	-2.316	0.880
2800	3	-0.848	-1.733	1.977	-1.152	-2.723	0.880
2825	4	-0.191	-1.690	2.002	-0.880	-2.909	0.936
2850	3	0.105	-1.733	2.079	-1.083	-2.197	0.880
2875	3	0.105	-1.733	2.045	-1.083	-2.661	0.871
2900	3	0.105	-1.733	2.079	-1.083	-2.233	0.889
2925	3	0.105	-1.733	2.000	-1.083	-2.233	0.925
2950	4	0.503	-1.690	1.920	-0.802	-2.600	0.892
2975	5	0.027	-1.667	1.973	-0.690	-2.459	0.950
3000	5	-0.255	-1.831	1.863	-0.747	-2.452	0.937
3025	4	0.170	-1.694	1.965	0.511	-2.601	0.884
3050	4	0.170	-1.690	2.002	0.511	-2.281	0.921
3075	3	-0.405	-1.733	2.079	0.378	-2.313	0.880
3100	4	-0.802	-1.690	2.002	0.401	-2.796	0.892
3125	5	-1.079	-1.682	1.933	0.424	-2.664	0.969
3150	5	-1.079	-1.682	1.840	0.424	-2.596	0.969
3175	6	-1.285	-1.667	1.736	0.447	-2.603	0.963
3199	5	-1.831	-1.682	1.886	0.309	-2.716	0.969
3217	6	-1.940	-1.810	1.906	0.339	-2.513	0.991

PPO1

Midpoint	S	D observed	Simulated D (0.025)	Simulated D (0.975)	H observed	Simulated H (0.025)	Simulated H (0.975)
125	16	2.353	-1.657	1.974	-0.385	-2.762	0.976
150	16	2.353	-1.676	1.853	-0.385	-2.138	0.986
175	19	2.130	-1.689	1.910	-0.768	-2.796	1.022
200	22	2.092	-1.640	1.833	-1.280	-2.367	0.996
225	21	1.934	-1.615	1.865	-1.255	-2.423	0.967
250	18	1.881	-1.599	1.723	-1.506	-2.571	0.977
275	20	1.845	-1.611	1.801	-1.753	-2.453	1.000
300	21	1.967	-1.737	1.936	-1.733	-2.291	0.990
325	19	1.903	-1.592	1.807	-1.262	-2.206	1.019
350	19	1.071	-1.692	1.935	-2.619	-2.335	0.987
375	18	0.972	-1.622	2.029	-2.919	-2.535	1.014
400	19	0.867	-1.664	1.972	-2.799	-2.382	1.026
425	17	0.999	-1.710	1.933	-2.393	-2.590	1.023
450	15	1.058	-1.665	1.979	-1.968	-2.413	0.966
475	16	0.904	-1.670	1.787	-1.871	-2.446	1.033
500	17	1.316	-1.630	1.981	-1.713	-2.595	1.007
525	17	0.935	-1.680	1.817	-1.256	-2.310	1.021
550	16	0.733	-1.749	1.949	-1.271	-2.677	1.031
575	16	0.733	-1.659	1.808	-1.271	-2.026	0.998
600	13	0.923	-1.536	1.802	0.093	-2.352	1.016
625	12	0.697	-1.641	1.898	0.833	-2.386	1.006
650	13	0.724	-1.677	1.850	-0.077	-2.390	0.985
675	13	0.741	-1.710	1.909	-0.487	-2.728	1.020
700	12	0.489	-1.600	1.738	-0.390	-2.573	0.997
725	11	0.659	-1.695	1.803	-0.456	-2.458	0.978
750	9	0.157	-1.621	1.923	-0.693	-2.373	1.014
775	7	0.337	-1.651	1.878	-1.074	-2.367	0.950
800	8	0.661	-1.709	2.011	-1.291	-2.275	0.993
825	8	0.661	-1.712	2.062	-1.291	-2.103	0.963
850	9	0.119	-1.626	1.995	-1.294	-2.524	0.962
875	9	-0.175	-1.697	2.000	-1.382	-2.542	0.995
900	8	-0.204	-1.776	2.034	-0.350	-2.363	0.981
925	7	-0.574	-1.689	1.820	-0.003	-2.499	0.968
950	7	-0.574	-1.693	1.817	-0.003	-2.194	0.974
975	7	-0.574	-1.719	1.795	-0.003	-2.572	0.957
1000	7	-0.574	-1.649	1.967	-0.003	-2.642	0.978
1025	8	-0.238	-1.665	1.855	0.287	-2.344	0.989
1050	8	-0.875	-1.661	1.948	0.577	-2.346	0.991
1075	8	-0.875	-1.609	1.787	0.577	-2.396	0.973
1100	7	-0.286	-1.693	1.855	0.693	-2.375	0.942
1125	5	0.126	-1.692	1.811	0.642	-2.691	0.953
1150	5	-0.231	-1.614	1.942	0.543	-2.520	0.948
1175	6	-0.550	-1.685	2.004	0.534	-2.557	0.960
1200	6	-0.550	-1.665	2.025	0.534	-2.864	0.960
1225	6	-0.550	-1.629	1.805	0.534	-2.781	0.975
1250	6	-0.550	-1.693	2.141	0.534	-2.718	0.970
1275	5	-0.491	-1.682	2.051	0.137	-2.706	0.926
1300	4	-0.110	-1.677	2.059	0.121	-2.748	0.907

1325	4	-0.110	-1.674	1.988	0.121	-2.291	0.906
1350	4	0.529	-1.677	2.004	-0.013	-2.378	0.907
1375	4	0.529	-1.674	1.896	-0.013	-2.592	0.913
1400	3	1.205	-1.578	2.037	-0.044	-2.513	0.869
1425	2	2.287	-1.456	2.114	-0.084	-2.844	0.852
1450	3	1.963	-1.578	2.109	0.114	-2.264	0.892
1475	6	1.192	-1.616	2.158	-0.125	-2.752	0.967
1500	6	1.192	-1.682	1.900	-0.125	-2.836	0.982
1525	8	1.646	-1.757	1.970	-0.313	-2.463	1.006
1550	8	1.646	-1.698	1.934	-0.313	-2.445	0.955
1575	11	1.611	-1.659	1.778	-0.106	-2.678	1.019
1600	10	1.368	-1.708	1.900	-0.129	-2.589	1.010
1625	11	1.049	-1.634	1.922	-0.106	-2.127	0.995
1650	12	0.989	-1.639	1.774	-0.003	-2.529	1.033
1674	13	0.965	-1.653	1.820	0.102	-2.430	1.012

PPO2

Midpoint	S	D observed	Simulated D (0.025)	Simulated D (0.975)	H observed	Simulated H (0.025)	Simulated H (0.975)
200	8	-1.035	-1.673	1.886	-4.859	-2.561	0.984
225	8	-1.035	-1.797	1.827	-4.859	-2.210	1.007
250	8	-1.035	-1.728	1.794	-4.859	-1.984	1.015
275	10	-1.017	-1.706	1.839	-5.282	-2.521	1.004
300	10	-1.105	-1.720	1.896	-5.313	-2.668	0.991
325	11	-0.950	-1.745	1.845	-4.993	-2.419	0.992
350	8	-0.828	-1.631	1.923	-3.036	-1.952	0.991
375	8	-0.938	-1.691	1.844	-2.578	-2.296	0.976
400	8	-0.832	-1.672	1.890	-2.454	-2.701	0.999
425	7	-0.735	-1.701	1.858	-2.661	-2.151	0.976
450	8	-0.186	-1.700	1.905	-2.474	-2.717	1.007
475	10	0.163	-1.636	2.007	-2.002	-2.529	1.031
500	10	0.163	-1.812	1.892	-2.002	-2.489	0.998
525	10	0.555	-1.693	1.869	-1.347	-2.455	1.004
550	9	0.857	-1.751	1.884	-1.444	-2.367	0.991
575	8	0.852	-1.640	1.844	-1.650	-2.226	0.992
600	8	0.852	-1.677	1.831	-0.568	-2.399	0.984
625	7	0.769	-1.819	1.919	-0.751	-2.505	0.993
650	7	0.769	-1.690	1.853	0.292	-2.539	0.993
675	7	0.769	-1.753	1.853	0.292	-2.911	1.010
700	6	0.272	-1.825	2.079	0.247	-2.672	0.948
725	5	-0.105	-1.709	1.904	0.082	-2.800	0.926
750	5	-0.105	-1.709	1.957	0.082	-2.481	0.948
775	6	0.255	-1.692	1.785	0.185	-2.593	0.938
800	7	0.035	-1.614	1.781	-0.787	-2.290	0.976
825	7	0.035	-1.722	1.945	-0.787	-2.496	0.950
850	7	0.035	-1.700	1.884	-0.787	-2.514	0.958
875	8	0.400	-1.746	1.854	-0.852	-2.531	0.984
900	7	0.636	-1.690	1.787	-0.977	-2.495	0.976
925	9	0.296	-1.726	1.935	-1.576	-2.931	0.998
950	9	0.296	-1.671	1.771	-1.576	-2.461	1.005
975	8	0.234	-1.691	1.956	-1.788	-2.420	0.992
1000	8	0.234	-1.718	1.874	-1.788	-2.124	0.968
1025	5	-0.471	-1.709	1.879	-2.018	-2.569	0.959
1050	6	-0.759	-1.691	1.952	-3.047	-2.960	0.967
1075	7	-0.371	-1.711	1.786	-2.733	-2.323	0.967
1100	9	0.715	-1.679	1.768	-2.709	-2.382	1.027
1125	11	0.639	-1.664	1.628	-2.509	-2.270	1.034
1150	12	0.395	-1.651	1.878	-2.387	-2.620	1.015
1175	10	0.666	-1.701	1.812	-1.907	-2.590	0.998
1200	10	0.666	-1.705	1.792	-1.907	-2.476	1.024
1225	10	0.666	-1.716	1.839	-1.907	-2.475	0.998
1250	11	0.824	-1.820	1.771	-1.713	-2.418	1.004
1275	11	0.824	-1.727	1.750	-1.713	-2.276	0.998
1300	9	1.381	-1.675	1.784	0.013	-2.710	0.991
1325	8	1.249	-1.709	1.997	-0.125	-2.382	0.999
1350	6	0.527	-1.825	1.859	0.216	-2.583	0.967
1375	4	-0.115	-1.707	1.807	0.525	-2.123	0.926

1400	3	0.437	-1.734	2.099	0.519	-2.384	0.887
1425	4	1.014	-1.707	1.917	0.471	-2.807	0.952
1450	4	1.014	-1.707	1.822	0.471	-2.445	0.913
1466	4	1.014	-1.707	1.846	0.471	-2.215	0.926

PPO3

Midpoint	S	D observed	Simulated D (0.025)	Simulated D (0.975)	H observed	Simulated H (0.025)	Simulated H (0.975)
176	12	0.625	-1.707	1.740	0.394	-2.311	1.019
201	10	0.981	-1.688	1.837	0.265	-2.423	1.018
226	7	1.590	-1.744	1.835	0.892	-2.730	0.961
251	6	0.601	-1.656	1.791	0.569	-2.566	0.970
276	6	0.601	-1.763	1.841	0.569	-2.526	0.971
301	6	0.601	-1.782	1.678	0.569	-2.474	0.997
326	6	0.601	-1.779	1.914	0.569	-2.460	0.984
351	4	0.538	-1.667	1.845	0.396	-2.520	0.920
376	3	0.094	-1.729	1.985	0.245	-2.593	0.858
401	3	0.094	-1.729	1.985	0.245	-2.595	0.900
426	2	0.541	-1.515	1.972	0.139	-2.659	0.802
451	2	0.541	-1.515	1.918	0.139	-2.766	0.802
476	2	-0.836	-1.515	1.919	0.139	-2.766	0.802
501	1	-0.641	-1.162	1.554	0.000	-3.153	0.622
526	2	-0.031	-1.515	1.972	0.232	-2.766	0.802
551	4	0.538	-1.667	1.859	0.396	-2.265	0.903
576	7	0.155	-1.772	1.778	-0.243	-2.147	0.984
601	8	-0.136	-1.742	1.866	-0.183	-2.466	1.040
626	10	-0.467	-1.678	1.891	-0.044	-2.396	0.991
651	10	-0.467	-1.688	1.789	-0.044	-2.360	1.027
676	10	-0.467	-1.731	1.934	-0.044	-2.414	1.018
701	10	-0.467	-1.769	1.713	-0.044	-2.342	1.018
726	10	-0.079	-1.700	1.907	0.062	-2.559	1.009
751	10	-0.079	-1.661	1.853	0.062	-2.306	0.991
776	9	-0.213	-1.730	1.797	-0.074	-2.672	0.988
801	7	-0.625	-1.679	1.807	-0.253	-2.479	0.984
826	4	-0.659	-1.667	1.845	0.384	-2.442	0.903
851	3	-0.233	-1.729	2.027	0.354	-2.831	0.900
876	2	1.882	-1.515	1.971	0.263	-2.656	0.802
901	3	1.999	-1.729	1.985	0.422	-3.150	0.900
926	3	1.999	-1.729	1.985	0.422	-2.400	0.900
951	3	1.999	-1.729	2.013	0.422	-2.596	0.857
976	2	1.757	-1.515	1.971	0.294	-2.575	0.802
1001	3	1.250	-1.729	1.985	-0.776	-2.593	0.879
1026	3	1.250	-1.729	1.985	-0.776	-2.079	0.879
1051	3	1.250	-1.729	2.189	-0.776	-2.272	0.879
1076	3	1.250	-1.729	1.795	-0.776	-2.080	0.900
1101	4	1.479	-1.667	2.000	-0.532	-2.550	0.903
1126	5	0.705	-1.809	1.794	-0.275	-3.092	0.940
1151	5	0.095	-1.809	1.859	-0.332	-2.381	0.955
1176	5	0.095	-1.809	1.868	-0.332	-2.955	0.940
1201	5	0.095	-1.809	1.793	-0.332	-2.440	0.940
1226	5	0.095	-1.809	1.793	-0.332	-2.440	0.940
1251	5	-0.064	-1.650	1.784	0.641	-2.470	0.970
1276	7	-0.387	-1.743	1.864	0.659	-2.491	0.973
1301	10	-0.666	-1.699	1.923	0.812	-2.343	1.009
1317	10	-0.666	-1.731	1.853	0.812	-2.387	1.000
